# Genetic epidemiology of *BRCA1- and BRCA2*-associated cancer across Latin America

**DOI:** 10.1038/s41523-021-00317-6

**Published:** 2021-08-19

**Authors:** Josef S. Herzog, Yanin Chavarri-Guerra, Danielle Castillo, Julio Abugattas, Cynthia Villarreal-Garza, Sharon Sand, Jessica Clague-Dehart, Rosa M. Alvarez-Gómez, Talia Wegman-Ostrosky, Alejandro Mohar, Pamela Mora, Azucena Del Toro-Valero, Adrian Daneri-Navarro, Yenni Rodriguez, Marcia Cruz-Correa, Patricia Ashton-Prolla, Bárbara Alemar, Rosa Mejia, Lenny Gallardo, Robin Shaw, Kai Yang, Aleck Cervantes, Kevin Tsang, Bita Nehoray, Hugo Barrera Saldana, Susan Neuhausen, Jeffrey N. Weitzel

**Affiliations:** 1 City of Hope, Duarte, CA USA; 2 Instituto Nacional de Ciencias Medicas y Nutrición, Salvador Zubiran, Mexico City, Mexico; 3 Instituto Nacional de Enfermedades Neoplásicas, Lima, Peru; 4 Hospital Zambrano Hellion TecSalud, Tecnologico de Monterrey, Monterrey, Mexico; 5 Instituto Nacional de Cancerología, México City, México; 6 Instituto de Investigaciones Biomédicas, Mexico City, Mexico; 7 Instituto Jalisciense de Cancerología, Centro Universitario de Ciencias de la Salud, Universidad de Guadalajara, México City, México; 8 Centro de Oncologia, Clínica del Country, Bogotá, Colombia; 9 University of Puerto Rico Comprehensive Cancer Center, San Juan, Puerto Rico; 10 Hospital de Clínicas de Porto Alegre and Universidade Federal do Rio Grande do Sul, Porto Alegre, Rio Grande do Sul Brazil; 11 Facultad de Medicina, Universidad Autónoma de Nuevo León, Monterrey, Mexico; 12 Latin American School of Oncology (Escuela Latinoamericana de Oncología), Tuxla Gutiérrez, Chiapas, Mexico; 13 Present Address: School of Community & Global Health, Claremont Graduate University, Claremont, CA USA; 14 Present Address: Roche Pharmaceutical, Basel, Switzerland

**Keywords:** Health sciences, Genetic testing, Diagnostic markers

## Abstract

The prevalence and contribution of *BRCA1*/*2* (*BRCA*) pathogenic variants (PVs) to the cancer burden in Latin America are not well understood. This study aims to address this disparity. *BRCA* analyses were performed on prospectively enrolled Latin American Clinical Cancer Genomics Community Research Network participants via a combination of methods: a Hispanic Mutation Panel (*HISPANEL*) on MassARRAY; semiconductor sequencing; and copy number variant (CNV) detection. *BRCA* PV probability was calculated using BRCAPRO. Among 1,627 participants (95.2% with cancer), we detected 236 (14.5%) BRCA PVs; 160 *BRCA1* (31% CNVs); 76 *BRCA2* PV frequency varied by country: 26% Brazil, 9% Colombia, 13% Peru, and 17% Mexico. Recurrent PVs (seen ≥3 times), some region-specific, represented 42.8% (101/236) of PVs. There was no ClinVar entry for 14% (17/125) of unique PVs, and 57% (111/196) of unique VUS. The area under the ROC curve for BRCAPRO was 0.76. In summary, we implemented a low-cost *BRCA* testing strategy and documented a significant burden of non-ClinVar reported *BRCA* PVs among Latin Americans. There are recurrent, population-specific PVs and CNVs, and we note that the BRCAPRO mutation probability model performs adequately. This study helps address the gap in our understanding of *BRCA*-associated cancer in Latin America.

## Introduction

Breast cancer is the most frequently diagnosed cancer and the leading cause of cancer-related mortality in Latin American women^1^. High mortality rates in the region are driven in part by advanced stage at diagnosis and limited access to cancer care^2^. Also, the mean age at diagnosis is younger than US non-Hispanic white (NHW) populations and there is a high prevalence of hormone receptor and Her2 negative breast cancer (TNBC), features common in hereditary disease. Thus, strategies aimed at preventing, or detecting breast cancer at an earlier stage, are crucial within the region.

Hereditary breast and ovarian cancer (HBOC) syndrome accounts for approximately 10% of all breast cancer and 15–20% of ovarian cancer cases, and the most commonly associated genes with HBOC are *BRCA1* and *BRCA2* (*BRCA*)^3^. Women with likely pathogenic and pathogenic variants (PVs) in either of these genes, have up to 80% lifetime risk of breast cancer and 20–50% risk of ovarian cancer^4^. The identification of PVs in at-risk individuals has significant clinical utility, as there are effective interventions aimed at both cancer prevention and early diagnosis^5^. We previously reported a high rate of *BRCA* PVs among 110 United States (U.S.) Hispanics with early-onset breast cancer^6^. However, Hispanics are not well represented in most studies^7,8^. For example, just 3.4% of participants were Hispanic in a recent study of female PV carriers from 69 centers in 49 countries on 6 continents^9^.

*BRCA* PVs are present in around 1/800 people in the general population. However, a higher rate of PVs has been reported in some founder populations such as the Ashkenazi Jewish, with a PV prevalence of 1/40^10^. From published high risk clinic series, approximately 10% of women meeting National Comprehensive Cancer Network (NCCN) genetic testing criteria will carry a *BRCA* PV^5,11^. Some studies suggest that individuals with Latin American or African ancestry have a higher prevalence of PVs^7^ while others demonstrated a lower yield among Hispanic immigrants in the United States (U.S.)^12^. Our group reported a high prevalence of *BRCA* PVs (25%) in a large study of Hispanics living in the Southwestern U.S.^13^.

The frequency of *BRCA* PVs has been reported to be between 1.2 and 15.6% among Latin American cancer patients^14–17^, and 15–28% among young breast or ovarian cancer patients in Mexico, unselected for family history of breast cancer^18,19^. However, the prevalence and contribution of *BRCA* PVs to the overall cancer burden in the region are not yet well understood.

Women with HBOC are usually younger at the time of breast cancer diagnosis, have a family history of breast and/or ovarian cancer and more frequently present with TNBC. Each of these characteristics is an indication for genetic testing according to international guidelines^20^. In Latin America, the proportion of young women with breast cancer is almost twice that seen in more developed regions of the world, and there also is a greater proportion of TNBC, meaning that a significant number of Latin American patients would meet criteria for gene testing^21,22^. Unfortunately, despite this great need, access to genetic cancer risk assessment and testing is limited across the region and is not covered by public insurance^23^.

Because genetic testing is expensive, pretest probability models are useful for selecting women at a higher risk of carrying a *BRCA* PV. However, because probability models are based mainly on data from NHW populations, their performance among Hispanics is limited. Some studies have found that BRCAPRO accuracy might be inferior in US minorities because the prevalence of carriers may differ by ethnicity and race^24^. BRCAPRO performance was reported as greater than 80% in NHW, 75% in African Americans and 58–75% in small samples of US Hispanics^25,26^.

*BRCA* PVs are detected by complex genetic technologies including multiple mutation scanning methods and Sanger DNA sequencing. Assays for the detection of large genomic rearrangements, herein referred to as copy number variants (CNVs), are also expensive. CNVs represented 11% of *BRCA* PVs among US Hispanics^13^, of which 62% were the *BRCA1* exon 9–12del (c.548-?_4185 + ?del), a Mexican founder PV, seen most frequently among patients from central Mexico, and estimated to have originated ~1,440 years ago^13,21,22,27^.

Commercial costs for these tests range between $249 and $5,000 U.S. dollars (USD), which is not affordable as an out-of-pocket expense for most of the Latin American population^28^. Disparities in access to testing have a significant impact on affected populations, due in part to underrepresentation in surveys of regional cancer etiology and in genomic variant databases^9,29–32^. To address disparities in genetic testing, we have developed and implemented a low-cost testing strategy for at-risk individuals, as part of a larger dissemination and implementation intervention^33^.

Here we report our results employing a sequential genetic testing strategy to detect *BRCA* PVs in a large sample of Latin Americans meeting referral criteria, using a combination of Sequenom MassARRAY technology, Ion Torrent semiconductor sequencing, and PCR-based methodologies for CNVs. We also report on the performance of the BRCAPRO mutation prediction model among our Latin American cohort.

## Results

### Accrual and study population characteristics

**Fig. 1:**
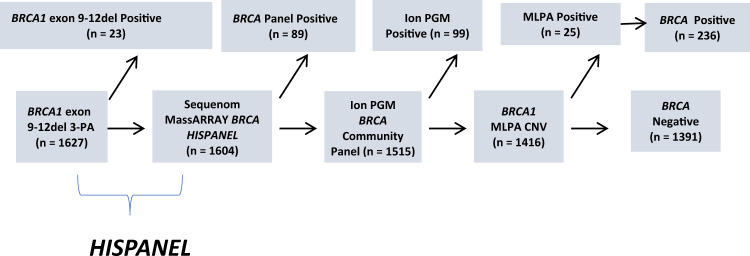
BRCA testing stratagem and pathogenic variant yield. A stepwise low-cost screening strategy was developed and implemented in our laboratory for the genotyping of a large Latin American cohort for mutations in the *BRCA* genes. Yield from each analysis and summary is noted above the respective sequential testing methods.

From December 2012 to August 2017, a total of 1627 participants were prospectively enrolled. Median age at enrollment was 44.6 years (range 14–96 years); and 1,607 (98.8%) were women. Of the 1627, 1375 (84.5%) had a primary breast cancer, 125 (7.7%) ovary cancer, and 49 (3.0%) other cancer types. Family history of breast cancer at ≤ 50 years in at least one first- or second-degree relative was reported in 412 (25.3%) participants, and of ovarian cancer at any age in 133 (8.2%) participants. Demographic characteristics are described in Table 1. The mean age at diagnosis of a first breast cancer in participants was 40.3 years (range 19–79); 15.0% had clinical stage 0-I and 85.0% had stage II-IV; 31.4% had TNBC (Table 2). A stepwise low-cost screening strategy (Fig. 1) was developed and implemented in our laboratory for the genotyping of a large Latin American cohort for mutations in the *BRCA* genes. Incremental genotyping costs by strategy was analyzed (Supplementary Table 1), and all strategies totaled approximately $100/case.

**Table 1:** Demographic characteristics.

	*N* = 1627 (%)
Median age (range) years	44.6 (14–96)
Female	1607 (98.8%)
Male	20 (1.2%)
Country of origin	
Peru	653 (40.1%)
Mexico	632 (38.9%)
Colombia	225 (13.8%)
Brazil	74 (4.6%)
Puerto Rico	43 (2.6%)
Disease status	
Affected (personal history of cancer)	1549 (95.2%)
Unaffected	78 (4.8%)
First cancer diagnosis	
Breast	1375 (84.5%)
Ovary/Fallopian tube	125 (7.7%)
Pancreas	3 (0.2%)
Prostate	5 (0.3%)
Melanoma	5 (0.3%)
Other cancers	36 (2.2%)
Second cancer diagnosis	
Breast (invasive and DCIS)	184 (11.3%)
Ovary/Fallopian tube	27 (1.7%)
Melanoma	6 (0.4%)
Other cancer	51 (3.1%)
Family history of cancer	
FDR with breast cancer < 50 y.o.	243 (15.0%)
SDR with breast cancer < 50 y.o.	169 (10.5%)
FDR with ovarian cancer	69 (4.3%)
SDR with ovarian cancer	64 (4.0%)

*FDR* first-degree relative, *SDR* second-degree relative, *y.o.* years old

**Table 2:** Breast cancer phenotype.

	*n* = 1375 (%)^a^
Mean age at first BC diagnosis (range) years	40.3 (19–79)
BC stage	
0	22 (3.5%)
I	73 (11.5%)
II	271 (42.7%)
III	230 (36.3%)
IV	38 (6.0%)
UNK	741
BC receptor status	
ER/PR positive	510 (37.1%)
ER/PR negative	299 (21.7%)
HER2 positive	149 (10.8%)
HER2 negative	632 (46.0%)
Triple negative	245 (17.8%)
UNK	594
Bilateral BC	61 (4.4%)

*BC* breast cancer, *ER* estrogen receptor, *PR* progesterone receptor, *UNK* unknown.

^a^Of those with known BC stage status (*n* = 634) or known BC receptor status (*n* = 781).

### Frequency of *BRCA* variants

The overall frequency of *BRCA* PVs for the cohort was 14.5% (236/1627). The population with the highest frequency was Brazil (25.7%), followed by Mexico (17.4%), and Peru (12.6%), while the lowest frequencies were found in Colombia and Puerto Rico (9.3%).

There were 196 unique variants of unknown significance (VUS). The VUS rate by country varied significantly: 12.2% for Brazil, 9.8% for Colombia, 12.2% for Mexico, 17.8% for Peru and 9.3% for Puerto Rico (*p* = 0.006).

Of the 125 unique PVs (Supplementary Data 1), 17 (13.6%) were not represented in ClinVar, a freely accessible, public archive of reports of the relationships among human genetic variations and phenotypes, with supporting evidence (https://www.ncbi.nlm.nih.gov/clinvar/). Two of those 17 were recurrent. Of the 196 unique variants of unknown significance (VUS), 111 (56.6%) were not in ClinVar (Supplementary Data 1).

### *HISPANEL* and ion torrent sequencing: sensitivity and performance

Primary screening with our custom designed recurrent Hispanic Mutation Panel (*HISPANEL*) comprised of 114 known PVs on the Sequenom MassARRAY platform, combined with a three-primer PCR assay (3-PA) for the Mexican founder PV (*BRCA1* exon 9–12del), yielded 47.4% (112/236) of all positives (Table 3). The *HISPANEL* sensitivity was 47% overall; 57% (63/110) in Mexico; 57% (12/21) in Colombia; 53% (10/19) in Brazil; 50% (2/4) in Puerto Rico; and 30% (25/82) in Peru. *BRCA* sequencing yielded 41.9% (99/236) of all positives and other CNV methods yielded 11% (23/236).

**Table 3:** *HISPANEL* and sequencing summary and yield by center.

Center	*n*	HISPANEL PVs	Non-HISPANEL Mutations	HISPANEL Sensitivity (Observed)	PV Frequency by Center
Bogotá, Colombia	225	12	9	0.57	0.09
Guadalajara, México	94	10	7	0.59	0.18
México City, México	538	53	40	0.57	0.17
Lima, Peru	653	25	57	0.30	0.13
San Juan, Puerto Rico	43	2	2	0.50	0.09
Porto Alegre, Brazil	74	10	9	0.53	0.26
TOTALS	1627	112	124	0.47	0.14

HISPANEL = Sequenom BRCA PV Panel (114 insertion/deletion or single nucleotide variants) and a PCR assay for the BRCA1 exon 9–12del CNV.

### Copy number variants

We found a total of 48 CNVs, all of them in *BRCA1*. No *BRCA2* CNVs were detected. *BRCA1* CNVs represented 20.3% (48/236) of all positives and 30% of *BRCA1* PVs, with the Mexican founder PV, *BRCA1* exon 9–12del (detected by investigator developed PCR assay^27^), accounting for 47.9% (23/48) of all CNVs. The remaining 52.1% of *BRCA1* CNVs were detected by NGS and confirmed by multiplex ligation-dependent probe amplification (MLPA). In Mexico, 34.5% (38/110) of all PVs were CNVs, followed by 9.5% in Colombia, 8.5% in Peru, and 5.3% in Brazil. In Puerto Rico 1 of 4 PVs was a CNV. Within Mexico, CNVs represented 41.2% (7/17) of PVs in Guadalajara (including one *BRCA1* exon 9–12del variant) and 33.3% (31/93) of PVs in Mexico City (including 22 *BRCA1* exon 9–12del CNVs).

### Recurrent PVs

Of the 236 PVs detected in this cohort, 101 (42.8%) were recurrent (observed 3 or more times) in the cohort (Table 4). The most frequent PV was the *BRCA1* exon 9–12del (c.548-?_4185 + ?del) PV, observed 23 times in Mexico. The Ashkenazi Jewish founder PV, *BRCA1* c.66_67delAG, was next (*n* = 13; 8 Peru, 5 Mexico), followed by *BRCA1* c.5123 C > A (*n* = 11), c.815_824dup (*n* = 8; 5 Peru, 3 Mexico), and another Ashkenazi Jewish founder PV, c.5266dupC (*n* = 8; 6 Brazil, 2 Mexico). PVs found to be frequent in Colombia include: *BRCA1* c.3331_3334del (*n* = 3; 2 Colombia, 1 Brazil) and c.5123 C > A (*n* = 11; 4 Colombia, 4 Mexico, 3 Peru). Regarding *BRCA2*, only a Colombian founder variant was recurrent: c.2808_2811del (*n* = 6: 3 in Brazil, 2 in Mexico and 1 in Colombia). In Brazil 3 recurrent PVs accounted for 52.6% (10/19) of the total; in Mexico 11 recurrent PVs accounted for 52.7% (58/110); in Peru 6 recurrent PVs accounted for 31.7% (26/82); in Colombia 3 recurrent PVs accounted for 33.3% (7/21).

**Table 4:** Distribution of recurrent (*n* ≥ 3) pathogenic variants.

GENE	Variant (HGVS)	Brazil	Colombia	Mexico	Peru	Puerto Rico	Total
BRCA1	c.548-?_4185 + ?del^H^	0	0	23	0	0	23
	c.66_67del (p.Glu23fs)^H^	0	0	5	8	0	13
	c.5123 C > A (p.Ala1708Glu)^H^	0	4	4	3	0	11
	c.815_824dup (p.Thr276fs)^H^	0	0	3	5	0	8
	c.5266dupC (p.Gln1756Profs)^H^	6	0	2	0	0	8
	c.−19-?_6325 + ?del^NC^	0	0	6	0	0	6
	c.4645_4646dup (p.Thr1550Lysfs)^NC^	0	0	0	4	0	4
	c.5075-?_5193 + ?del	0	0	4	0	0	4
	c.122 A > T (p.His41Leu)	0	0	3	0	0	3
	c.3331_3334del (p.Gln1111fs)^H^	1	2	0	0	0	3
	c.5278-?_5467 + ?del	0	0	0	3	0	3
BRCA2	c.2808_2811del (p.Ala938Profs)^H^	3	1	2	0	0	6
	c.1219 C > T (p.Gln407Ter)	0	0	0	3	0	3
	c.3264dupT (p.Gln1089Serfs)^H^	0	0	3	0	0	3
	c.5631del (p.Asn1877fs)	0	0	3	0	0	3
Recurrent PVs (% of total by country)	10 (52.6)	7 (33.3)	58 (52.7)	26 (31.7)	0	101 (42.8)	

*PVs* pathogenic variants, *NC* Not in ClinVar, *H* on HISPANEL.

### BRCAPRO probability model

**Fig. 2:**
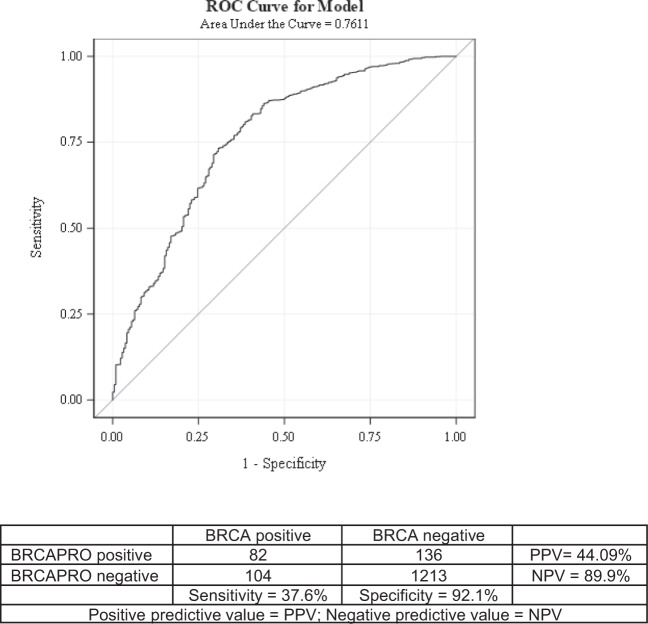
Area under the receiver operating characteristic (ROC) curve model for BRCAPRO. Performance of BRCAPRO mutation risk predictive model using ROC curve and AUC for this cohort is shown at the top, and the mutation carrier sensitivity, specificity, positive predicted value and negative predicted value is depicted at the bottom.

Sensitivity, specificity, positive predictive value (PPV) and negative predictive value (NPV) were calculated for the BRCAPRO model at a 10% threshold. The area under the receiver operating characteristic (AUROC) curve was 0.76 for BRCAPRO to predict the probability that a participant in this cohort carried a *BRCA* PV (Fig. 2). The sensitivity, specificity, PPV, NPV and accuracy were 37.6%, 92.1%, 44.1%, 89.9% and 84.3%, respectively.

## Discussion

This is the largest study of *BRCA* PVs among Latin Americans to date, and represents a cross-section including Brazil, Colombia, Peru, Puerto Rico and Mexico. Among 1,627 prospectively accrued participants, most affected with cancer, we detected 236 (14.5%) *BRCA* PVs using a low-cost *BRCA* genotyping workflow. Most previous studies were population specific and/or had a small sample size (Supplementary Data)^17^.

The prevalence of PVs was 9.3–25.7% across the countries included in this study, similar to the range (3.0–47.8%) reported in previous studies (Supplementary Data 2). There were wide variations in reported prevalence within each country. For example, a study of 327 patients in Mexico only reported 7.3% PVs^34^ and did not test for CNVs other than the Mexican founder PV. Another study of 101 patients in Mexico at high-risk and/or with breast cancer at or under the age of 40 years, which included CNV analysis, reported 13.9% PVs^35^, which more closely approximated our observation of 17% of the 632 patients in Mexico.

This study highlights the extent to which *BRCA* CNVs play an important role in the etiology of cancer in Latin America, and particularly in Mexico where CNVs represented 34.5% of PVs, nearly half of which were the Mexican founder, *BRCA1* exon 9–12del. The burden of this PV has previously been documented by us and others in independent Mexican cohorts^18,19,34,36^, and among Mexican Americans^13,27,37^. CNVs represented < 10% of PVs in Colombia, Peru, and Brazil. The sample size was too small to estimate for Puerto Rico.

The absence of *BRCA2* CNVs was notable. The Consortium of Investigators of Modifiers of BRCA1/2 (CIMBA) reported a ratio of 3.5:1 excess of *BRCA1* CNVs compared to *BRCA2*, among 18,435 breast cancer cases with *BRCA1* PVs and 11,351 with *BRCA2*, ascertained from 69 centers in 49 countries on 6 continents. However, Hispanics only represented 3.4% of the total which was primarily NHW^9^. We confirmed all CNVs by MLPA or long-range PCR, and the CNV detection algorithm incorporated into the Ion Reporter workflow for NGS performs well^38^. Two of the three studies in Mexico that included CNV analyses (Supplementary Data 2) did not detect any *BRCA2* CNVs, though 14 *BRCA1* CNVs were detected (11 of which were the Mexican founder PV)^35,36^. The remaining study reported 3 *BRCA1* CNVs and 11 *BRCA2* CNVs. However, 9 of 11 were single exon deletions and were not confirmed by a secondary MLPA kit or long-range PCR^39^, and confirmation is recommended due to a high false-positive rate for single exon CNVs on MLPA. Consequently, we believe the weight of evidence suggests a paucity of *BRCA2* CNVs among the populations studied here.

Although limited by high-risk clinic ascertainment, the observed frequency of *BRCA* PVs highlights the burden of hereditary cancer in every country we examined. We found the highest frequency of PVs in Brazil, where the inclusion criteria required a greater clinical burden of breast/ovarian cancer, followed by Mexico and Peru, while the lowest frequency was found in Colombia and Puerto Rico. Another limitation is known heterogeneity in genetic ancestral composition of among Hispanics from the Americas^40,41^. We found that the founder Jewish PVs (BRCA1 c.66_67del and c.5266dupC) were recurrent in our cohort. We were among the first to document a shared genotype between Ashkenazi Jewish individuals and Mexican Americans with no known Jewish ancestry who carried *BRCA1* c.66_67del^6^. Subsequent publications have reinforced the observation, and suggest they are likely descendants of conversos or crypto-Jews who emigrated to the Americas in the late 15th century and over generations assimilated into the larger Hispanic society, representing an underappreciated diaspora to the new world^13,42,43^.

In addition, we evaluated the BRCAPRO model performance for the first time in a Latin American cohort and found that it performed well (AUROC 0.76) and was comparable to that reported for genetic risk clinics in England (0.76)^44^. BRCAPRO can be used in clinical practice to select patients at risk for carrying *BRCA* PVs, though it was developed using rates derived from primarily European populations. Similar to our findings, a small study of U.S. Hispanics reported an AUROC of 0.70^26^. The BRCAPRO results from our study of native Latin American populations, reinforces our observations of prevalence and relationship to clinical phenotype, and also serve as a surrogate indication that our genetic testing methods were sensitive. Further, our study indicates that BRCAPRO can be used as a tool to select individuals at the highest risk of carrying *BRCA* PVs, which could enable prioritized allocation of limited resources in Latin America.

Worldwide, *BRCA* testing in the past was commonly performed by capillary DNA (Sanger) sequencing, which was considered the gold standard, albeit expensive. Our study spans 2012–2017, during which time we developed and applied inexpensive approaches, including the *HISPANEL* on a Sequenom MassARRAY spectroscopy platform, and subsequently, multiplex amplicon library NGS on the Ion Torrent PGM platform that could be deployed in countries with limited resources. The *HISPANEL* served well in early studies, with an analytic sensitivity compared to full sequencing of *BRCA1* and *BRCA2* of up to 70% in Mexico, largely because recurrent PVs constituted a large proportion of the overall burden. *HISPANEL* also performed well in Colombia, due to the prevalence of founder PVs in this country. For example, the three prevalent PVs seen in Colombia, *BRCA1* c.5123 C > A, *BRCA1* c.3331_3334del, and *BRCA2* c.4889 C > G, accounted for a third of all PVs found in this region. In Mexico, the *BRCA1* exon 9–12del PV accounted for a fifth of all mutations detected and was not seen outside of Mexico^45^. In Peru, the observed sensitivity of *HISPANEL* was 30%, approximately half of what was observed in Colombia and Mexico. This is consistent with the isolation of the Indigenous American Peruvian population, while other populations show more migration between regions^46^. Despite limitations, this report shows that using a low-cost strategy for broad access to *BRCA* genotyping in Latin America is feasible. The most recent iteration in our laboratory is a multiplex amplicon multigene panel, validated in the CARRIERS project^47^.

There are 125 unique PVs among the 236 *BRCA* PVs reported here; 17/125 (13.6%) were not represented in ClinVar, highlighting the underrepresentation of Latin American ancestry. This lack of representation may influence the effectiveness of variant curation by clinicians. Data from this ongoing study also are valuable to construct population-specific variant frequencies, and to increase representation in public databases^48^. We also found that 111/196 (56.6%) unique VUS were not reported previously in ClinVar. We recently demonstrated that sequence data well controlled for racial/ethnic composition can yield important observations about pathogenicity of uncertain variants^29^. The proportion of VUS reported varied by study and gene. The proportion of VUS that we report here (14%) is higher than that in NHW women and comparable to other studies including Hispanics^7,49,50^. In a meta-analysis of largely European populations, *BRCA1* VUS were reported in 1.23% of cases (95% CI 0.75–2.04) and for *BRCA2* in 3.29% of cases (CI 95% 0.75–2.42)^51^. Similarly, the frequency of VUS is elevated in other underrepresented populations, such as in African American and Asiatic^49,52^, and we demonstrated varying rates of variant reclassification by ancestry over time^50^. Again, increased representation in public databases is important for clinical translation in diverse populations. Over half of the VUS were not seen in ClinVar, suggesting there may be a registration bias towards deposit of PVs in ClinVar.

In conclusion, strategies to develop affordable *BRCA* testing in low- and middle-income countries with limited resources are important to extend the reach of lifesaving genetic cancer risk assessment (GCRA). The burden of hereditary breast and ovarian cancer in the respective countries is evident from this and other studies. More than 80% of the breast cancers in this series were diagnosed at Stage II or greater, and 268 cases had multiple primary tumors, including 27 ovarian cancers that might have been prevented if genetic testing was available at breast cancer diagnosis. Dissemination and implementation of GCRA, supported by clinical training and accessible genetic testing^33^, could result in measurable prevention of breast cancer and its related mortality in Latin America and other low- and middle-income countries globally.

## Methods

### Study population

Patients seen for GCRA through Clinical Cancer Genomics Community Research Network (CCGCRN)^13,53^ sites in Latin America between December 2012 and August 2017 were prospectively enrolled after informed consent on an IRB-approved protocol and offered genetic testing. The overall protocol was approved by the City of Hope IRB (#96144; ClinicalTrials.gov Identifier: NCT04185935) data coordinating center, and approved separately, as a Federated consortium at each participating center: (1) Instituto Nacional de Cancerlogía (INCan) in Mexico City, Mexico; (2) Instituto Jalisciense de Cancerologia, in Guadalajara, Mexico; (3) Instituto des Enfermedades Neoplásicas (INEN) in Lima, Peru; (4) Clínica del Country, Oncology Center, in Bogota, Colombia; (5) The University of Puerto Rico and MD Anderson Cancer Center in San Juan, Puerto Rico; and (6) Hospital de Clínicas de Porto Alegre in Porto Alegre, Brazil. Patients met the National Comprehensive Cancer Network (NCCN) guidelines for genetic/familial high-risk assessment: breast and ovarian^20^. Demographic characteristics, clinical variables and four-generation pedigrees focused on family cancer history were obtained. When more than one person was enrolled and tested in a family, the first person tested was selected for inclusion in study.

### DNA extraction and *BRCA* testing

Genomic DNA was obtained from peripheral blood drawn into ACD-A collection tubes using the Qiagen (Germantown, MD) FlexiGene DNA Kit.

### *BRCA1* exon 9–12del three-primer PCR assay (3-PA)

For all individuals, 25 ng of genomic DNA was screened for the presence of the copy number variant (CNV), *BRCA1* exon 9–12del (c.548-?_4185 + ?del), by a three-primer PCR assay (3-PA)^27^.

### Sequenom MassARRAY genotyping

As described previously, a panel of 114 known pathogenic recurrent *BRCA* point^6,27,13,54^ and insertion/deletion PVs among Hispanics was used to genotype all individuals on the Sequenom (San Diego, CA) MassARRAY platform, and together with the *BRCA1* exon 9–12del 3-PA, comprises our Hispanic mutation panel (*HISPANEL)*^19^.

### *BRCA* CNV

Using Ion Torrent PGM semiconductor sequencing (see below) a CNV baseline was established within the Ion Reporter workflow to enable detection of *BRCA1* and *BRCA2* CNV. Cases with uninformative *HISPANEL* and *BRCA* sequencing were further analyzed for CNVs in *BRCA1* by MLPA (MRC-Holland, Amsterdam, the Netherlands). For each MLPA reaction, 125 ng of DNA was PCR amplified with probemix P002 according to the manufacturer’s instructions. PCR fragments were size separated on an ABI (Applied Biosystems, Carlsbad, CA) 3130xl capillary sequencer. Dosage calculations and determination of CNV were performed with Coffalyser.Net software v.140701 (MRC-Holland) and/or GeneMarker software (SoftGenetics, State College, PA). CNVs detected by NGS or MLPA, were confirmed with MLPA probe mixes P087 and P045, respectively. *BRCA2* was only evaluated for CNV by NGS, in part due to the known 5-fold greater rate of occurrence in *BRCA1* relative to *BRCA2* of CNVs^37^.

### Ion torrent PGM semiconductor sequencing

*HISPANEL* negative cases that met NCCN criteria^20^ received full sequencing of all *BRCA* coding exons and exon-intron junctions on the Ion Torrent PGM platform. 10 ng of Qubit (Thermo Fisher Scientific, Waltham, MA) quantified DNA was PCR amplified using the commercially available Ion AmpliSeq *BRCA1* and *BRCA2* Community Panel (https://www.thermofisher.com/us/en/home/life-science/sequencing/next-generation-sequencing/ion-torrent-next-generation-sequencing-workflow/ion-torrent-next-generation-sequencing-select-targets/ampliseq-target-selection/community-panels.html) - pilot tested by our laboratory for the AmpliSeq *BRCA* Global Consortium^55^ – to generate targeted, barcoded, amplicon libraries. Following template preparation and enrichment, 36 individual sample libraries were loaded on an Ion 318 Chip v2 and run for 500 cycles (250 bp single end reads).

### Ion reporter cloud

Base calling, call qualification, sequence alignment (genome reference hg19), and coverage analysis were performed in Ion Torrent Suite 5.0.4 (Thermo Fisher Scientific). The average coverage was ≥ 500x across the 171 primers in the panel. After signal processing with Torrent Variant Caller, sequence aligned files were uploaded to Ion Reporter v5.0 Cloud (https://ionreporter.thermofisher.com/ir/) for additional variant calling, annotation and reporting. Using a custom designed workflow, with a CNV baseline established by sequencing 10 *BRCA* CNV negative control cases, individual cases were examined for all variants.

### Variant classification

All variants annotated in Ion Reporter were visualized in ClinVar (https://www.ncbi.nlm.nih.gov/clinvar/) for classification (last time accessed May 2019). Only entries from established commercial vendors (Ambry Genetics, Color Genomics, Counsyl, GeneDx, Invitae, and/or Myriad Genetics (through the Sharing Clinical Reports Project (SCRP), http://clinicalgenome.org/data-sharing/sharing-clinical-reports-project-scrp/), were considered. If a *BRCA* variant had been evaluated by the Evidence-based Network for Interpretation of Germline Mutant Alleles (ENIGMA) consortium (https://enigmaconsortium.org/), recognized as an expert reviewer in ClinVar, that classification was used. Variant call format files for variants without ClinVar entrees were also uploaded into Ingenuity Variant Analysis (IVA) (Qiagen, Germantown, MD) which applies American College of Medical Genetics and Genomics (ACMG) guidelines to variant classification.

### Variant confirmation

Small sequence variants (point, in/del PVs) were confirmed with Sanger sequencing using the BigDye Terminator v3.1 Cycle Sequencing Kit (Thermo Fisher Scientific) and run on an ABI (Applied Biosystems) 3130xl capillary sequencer. CNV variants detected by Ion Torrent sequencing were confirmed with MLPA (see above).

### Testing cost

The cost for each test was calculated in USD and based on laboratory reagents and materials as follows: *BRCA1* exon 9–12del 3-PA ($0.25/case), Sequenom MassARRAY *BRCA* Panel ($10/case), Ion Torrent PGM AmpliSeq *BRCA* Panel ($82/case) and *BRCA1* MLPA ($25/case).

### Mutation probability model analysis

The probabilities of carrying a *BRCA* PV were estimated using BRCAPRO (version 2.1.4) in 1,535 cases. BRCAPRO risk estimates are derived through a Bayesian probability model, and accounts for the patient’s first- and second-degree relatives, age of diagnosis of breast and/or ovarian cancer, and ages of unaffected family members^56^. Pedigrees were created electronically using Progeny (Progeny Software, Delray Beach, FL)^57^. Sensitivity, specificity, PPV and NPV were calculated for the BRCAPRO model at a 10% threshold. Calibration of the model was conducted by comparing observed versus predicted numbers of PV carriers. The AUROC was calculated to evaluate model discrimination.

### Statistical analysis

Variables were summarized using descriptive statistics, including mean, range and frequency. Frequencies of *BRCA* variants (pathogenic/VUS) were described for the whole cohort, and individually for each country. We also describe the type of mutation (CNV, recurrent and unique variants). Differences between countries of origin were compared and expressed as percentages. The sensitivity of *HISPANEL* testing was calculated using observed versus detectable, with full *BRCA* sequencing as the country-specific reference for testing. SAS version 9.4 analytic software (SAS Institute, Cary, NC) was used to carry out the statistical analysis.

### Reporting summary

Further information on research design is available in the Nature Research Reporting Summary linked to this article.

## Supplementary information

Supplementary Data 1

Supplementary Data 2

Reporting Summary

Supplementary Information

## Data Availability

The data generated and analyzed during this study are described in the following data record: 10.6084/m9.figshare.14910096^48^. The pathogenic BRCA variants and VUS data are included as separate tabs in ‘Supplementary tables 2 and 3.xlsx’, which is available via the online version of the related article as well as in Excel format as part of the data record. Unique variants have been deposited in *ClinVar* under accessions https://identifiers.org/clinvar.submission:SCV001739435 to https://identifiers.org/clinvar.submission:SCV001739444^58^. The minimum dataset necessary to interpret, replicate and/or build upon our methods will be made available on request to the corresponding author.
