# Supplementary material for: Genetic epidemiology of BRCA1- and BRCA2-associated cancer across Latin America
Source: NPJ Breast Cancer. 2021 Aug 19;7:107. doi: 10.1038/s41523-021-00317-6 (PMC8377150; doi:10.1038/s41523-021-00317-6)
Supplement: Supplementary file 3 — Reporting Summary [file 41523_2021_317_MOESM3_ESM.pdf]

nature research

Corresponding author(s): Jeffrey N. Weitzel, MD

Last updated by author(s): Dec 21, 2020

Reporting Summary

Nature Research wishes to improve the reproducibility of the work that we publish. This form provides structure for consistency and transparency in reporting. For further information on Nature Research policies, see our Editorial Policies and the Editorial Policy Checklist.

Statistics

For all statistical analyses, confirm that the following items are present in the figure legend, table legend, main text, or Methods section.

n/a	Confirmed	
☐	☒	The exact sample size (n) for each experimental group/condition, given as a discrete number and unit of measurement
☒	☐	A statement on whether measurements were taken from distinct samples or whether the same sample was measured repeatedly
☐	☒	The statistical test(s) used AND whether they are one- or two-sided<br>*Only common tests should be described solely by name; describe more complex techniques in the Methods section.*
☒	☐	A description of all covariates tested
☒	☐	A description of any assumptions or corrections, such as tests of normality and adjustment for multiple comparisons
☒	☐	A full description of the statistical parameters including central tendency (e.g. means) or other basic estimates (e.g. regression coefficient) AND variation (e.g. standard deviation) or associated estimates of uncertainty (e.g. confidence intervals)
☒	☐	For null hypothesis testing, the test statistic (e.g. F, t, r) with confidence intervals, effect sizes, degrees of freedom and P value noted<br>*Give P values as exact values whenever suitable.*
☒	☐	For Bayesian analysis, information on the choice of priors and Markov chain Monte Carlo settings
☒	☐	For hierarchical and complex designs, identification of the appropriate level for tests and full reporting of outcomes
☒	☐	Estimates of effect sizes (e.g. Cohen's d, Pearson's r), indicating how they were calculated

Our web collection on statistics for biologists contains articles on many of the points above.

Software and code

Policy information about availability of computer code

Data collection	No software was used.
Data analysis	SAS version 9.4 analytic software (SAS Institute, Cary, NC)<br>BRCAPRO (version 2.1.4)

For manuscripts utilizing custom algorithms or software that are central to the research but not yet described in published literature, software must be made available to editors and reviewers. We strongly encourage code deposition in a community repository (e.g. GitHub). See the Nature Research guidelines for submitting code & software for further information.

Data

Policy information about availability of data

All manuscripts must include a data availability statement. This statement should provide the following information, where applicable:

- Accession codes, unique identifiers, or web links for publicly available datasets
- A list of figures that have associated raw data
- A description of any restrictions on data availability

The minimum dataset necessary to interpret, replicate and/or build upon our methods will be made available on request, and unique variants will be submitted to the publicly accessible ClinVar database.

Field-specific reporting

Please select the one below that is the best fit for your research. If you are not sure, read the appropriate sections before making your selection.

☒ Life sciences ☐ Behavioural & social sciences ☐ Ecological, evolutionary & environmental sciences

For a reference copy of the document with all sections, see nature.com/documents/nr-reporting-summary-flat.pdf

Life sciences study design

All studies must disclose on these points even when the disclosure is negative.

Sample size	We selected the largest possible sample for analyses based on prospective accrual to a registry protocl
Data exclusions	No exclusions.
Replication	Sanger resequencing (and MLPA in the case of CNVs) was employed to validate all variants detected from the various approaches detailed in the methods section.
Randomization	Not relevant to this study.
Blinding	Blinding not relevant to this prospective study of the prevalence of BRCA variants in specific underserved populations.

Reporting for specific materials, systems and methods

We require information from authors about some types of materials, experimental systems and methods used in many studies. Here, indicate whether each material, system or method listed is relevant to your study. If you are not sure if a list item applies to your research, read the appropriate section before selecting a response.

Materials & experimental systems

n/a	Involved in the study	
☒	☐	Antibodies
☒	☐	Eukaryotic cell lines
☒	☐	Palaeontology and archaeology
☒	☐	Animals and other organisms
☐	☒	Human research participants
☐	☒	Clinical data
☒	☐	Dual use research of concern

Methods

n/a	Involved in the study	
☒	☐	ChIP-seq
☒	☐	Flow cytometry
☒	☐	MRI-based neuroimaging

Human research participants

Policy information about studies involving human research participants

Population characteristics	atients seen for GCRA through Clinical Cancer Genomics Community Research Network (CCGCRN) 13,46 sites in Latin America between December 2012 and August 2017 were prospectively enrolled after informed consent on an IRB-approved protocol and offered genetic testing. Six Latin American cancer centers were included: 1) Instituto Nacional de Cancerlogía (INCan) in Mexico City, Mexico; 2) Instituto Jalisciense de Cancerologia, in Guadalajara, Mexico; 3) Instituto des Enfermedades Neoplásicas (INEN) in Lima, Peru; 4) Clínica del Country, Oncology Center, in Bogota, Colombia; 5) The University of Puerto Rico and MD Anderson Cancer Center in San Juan, Puerto Rico; and 6) Hospital de Clínicas de Porto Alegre in Porto Alegre, Brazil. Patients met the National Comprehensive Cancer Network (NCCN) guidelines for genetic/familial high-risk assessment: breast and ovarian. Demographic characteristics, clinical variables and four-generation pedigrees focused on family cancer history were obtained. When more than one person was enrolled and tested in a family, the first person tested was selected for inclusion in study.
Recruitment	Guidelines based referral population. Comparable to other clinic based studies.
Ethics oversight	The overall protocol was approved by the IRB at City of Hope (#96144) data coordinating center, and approved separately, as a Federated consortium at each participating center.

Note that full information on the approval of the study protocol must also be provided in the manuscript.

Clinical data

Policy information about clinical studies

All manuscripts should comply with the ICMJE guidelines for publication of clinical research and a completed CONSORT checklist must be included with all submissions.

Clinical trial registration	ClinicalTrials.gov Identifier: NCT04185935
Study protocol	Clinical Trials.gov, or from authors upon request.
Data collection	Accrual December 2012 and August 2017; demographic characteristics, clinical variables and four-generation pedigrees focused on family cancer history were obtained. When more than one person was enrolled and tested in a family, the first person tested was selected for inclusion in study.
Outcomes	Prevalence of BRCA PVs and performance assessments of BRCAPRO model were pre-specified endpoints.
