# Supplementary material for: Genetic epidemiology of BRCA1- and BRCA2-associated cancer across Latin America
Source: NPJ Breast Cancer. 2021 Aug 19;7:107. doi: 10.1038/s41523-021-00317-6 (PMC8377150; doi:10.1038/s41523-021-00317-6)
Supplement: Supplementary file 4 — Supplementary Information [file 41523_2021_317_MOESM4_ESM.pdf]

Supplementary Table 1. Genotyping cost analysis

n	BRCA1 exon9-12del	Sequenom BRCA Panel	Ion Torrent BRCA Panel	BRCA1 MLPA	TOTALS	Cost/Case
23	$5.75				$5.75	$0.25
89	$22.25	$890.00			$912.25	$10.25
99	$24.75	$990.00	$8,118.00		$9,132.75	$92.25
25	$6.25	$250.00	$2,050.00	$625.00	$2,931.25	$117.25
1391	$347.75	$13,910.00	$114,062.00	$34,775.00	$163,094.75	$117.25
1627	$406.75	$16,040.00	$124,230.00	$35,400.00	$176,076.75	$108.22

Supplementary Data 1: Complete list of variants in *BRCA1* and *BRCA2*, with notation of number of observations and whether present in ClinVar database

Supplementary Data 2: Published Latin American *BRCA* studies for comparison to the current study
